# Fact-finding survey on the competencies and literacy of radiological technologists regarding radiation disasters

**DOI:** 10.3233/XST-221341

**Published:** 2023-03-15

**Authors:** Tomohiro Arai, Syo Murata, Yuichi Watanabe, Toshihiro Ishihara, Yoshiya Fukamizu, Satoshi Takeda, Kiyokadzu Ebata, Yuki Watanabe, Yoshio Takashima, Junichi Kaneko

**Affiliations:** a Faculty of Health Sciences, Komazawa University, Tokyo, Japan; b Department of Radiological Technology, National Cancer Center Hospital, Tokyo, Japan; c Department of Radiology, National Hospital Organization, Tokyo Medical Center, Tokyo, Japan; d Department of Radiology, National Hospital Organization, Sagamihara National Hospital, Sagamihara, Japan; e The Japan Association of Radiological Technologists, Tokyo, Japan; f Nuclear Fuel Cycle Engineering Laboratories, Japan Atomic Energy Agency, Ibaraki, Japan; g National Institute of Radiological Sciences, National Institutes for Quantum Science and Technology, Chiba, Japan

**Keywords:** Radiological technologist, risk communicator, radiation disaster

## Abstract

**BACKGROUND:**

Radiological technologists serve as risk communicators who aim to lessen patients’ anxiety about radiation exposure, in addition to performing radiological examinations.

**OBJECTIVE:**

We conducted a fact-finding survey on knowledge and awareness of radiation disasters among the radiological technologists to reveal their literacy and competencies regarding radiation disasters.

**METHODS:**

A paper questionnaire was distributed to 1,835 radiological technologists at 166 National Hospital Organization facilities in Japan. The 28-item questionnaire covered knowledge and awareness of radiation protection and radiation disasters. Radiological technologists were divided into 2 groups by regionality: areas where a nuclear power station was present/nearby (NPS areas) and non-NPS areas.

**RESULTS:**

Completed questionnaires were returned from 148 facilities with a facility response rate of 89.2% and from 1,391 radiological technologists with a response rate of 75.8%. There were 1,290 valid responses with a valid response rate of 70.3%. The correct answer rate for knowledge of radiation protection and radiation disasters was high in the 24 NPS areas. There were no differences in awareness of radiation disasters between NPS and non-NPS areas.

**CONCLUSIONS:**

Establishing a nationwide, region-independent training system can be expected to improve literacy regarding radiation disasters among radiological technologists. Willingness to assist during disasters was high among radiological technologists irrespective of area, indicating that the competencies of radiological technologists represent a competency model for radiation disaster assistance.

## 1 Background

Radiological technologists have received specialized education about radiation [1, 2], and serve as risk communicators who aim to lessen patients’ anxiety about radiation exposure, in addition to performing radiological examinations in routine clinical practice [3– 5]. Japan has a history of radiation-induced damage: the atomic bombs dropped in August 1945, the JCO criticality accident in Tokai-mura in September 1999 [6, 7], and the nuclear disaster at the Fukushima Daiichi Nuclear Power Plant operated by Tokyo Electric Power Company in March 2011 (hereinafter, referred to as the Fukushima nuclear accident). The major radionuclides released as a result of the Fukushima nuclear accident were ^131^I and ^137^Cs, and the estimated amount of these radionuclides released was equivalent to 10– 20% of that released as a result of the accident at the Chernobyl Nuclear Power Station [8]. In Fukushima Prefecture, radiological protection measures, such as evacuation and sheltering, were implemented in an area as wide as 1800 km^2^, and radiological technologists across Japan were dispatched to the affected area to conduct an essential procedure— screening the belongings and body surfaces of evacuees for contamination [9]. Furthermore, dispatched radiological technologists contributed to reducing the anxiety of local residents by providing reliable information on the health effects of radiation exposure [9, 10].

Out of 47 areas in Japan, there are 24 areas where a nuclear power station is present or nearby (hereinafter, called nuclear power station [NPS] areas). These areas have a medical system in place for nuclear emergencies [11], but the remaining 23 areas (non-NPS areas) do not because of restrictions in the accounting system of the national budget, even though urban areas such as Tokyo have enough healthcare staff. Fading awareness of radiation accidents over time is another concern [12, 13]. It is also important that persons with a certain level of radiation knowledge be dispatched to provide the assistance during a radiation disaster [14].

## 2 Objective

We conducted a fact-finding survey on knowledge and awareness of radiation disasters among radiological technologists at National Hospital Organization, which has medical facilities throughout Japan, to reveal their literacy and competencies regarding radiation disasters. Also, we compared the knowledge and awareness of radiation disasters among Japanese radiological technologists between NPS areas and non-NPS areas and discuss ideal human resource development for radiological technologists to be ready to serve during a radiation disaster.

## 3 Methods

This study was approved by the Ethics Review Board of Komazawa University (approval number: 21-27) and the Ethics Review Board of National Cancer Center Japan (approval number: 2021-454). A paper questionnaire survey was administered to 1,835 radiological technologists at 166 National Hospital Organization facilities between August 26 and September 9, 2022. Data were anonymized to prevent personally identified information from being linked to answers. The purposes of data use were explained on the consent form, and responses with the signed consent form were considered valid. No compensation was offered to the respondents before or after the survey, and no reminder was sent after distribution of the survey. The questionnaire comprised 28 questions on knowledge and awareness of radiation protection and radiation disasters (for the original questionnaire, see the electronic supplementary material). Radiological technologists were divided into 2 groups by regionality into an NPS area group and a non-NPS area group, and questionnaire results were analyzed by comparing these groups.

The detailed structure of the questionnaire was as follows. The content of the questionnaire was prepared based on materials published by the Japanese government [3, 10]. Questions 1– 5 covered basic knowledge of radiation protection, and questions 6– 11 covered basic knowledge of radiation disasters. All questions were multiple choice, and respondents chose a single answer from 5 possible answers (which included “Not sure”). Questions 12 and 13 asked the respondent’s age and sex, respectively, and questions 14– 18 covered opinions on and experience of disaster assistance activities. Radiological technologists who worked in an NPS area after Fukushima nuclear accident in 2011 were extracted and assigned to the NPS area group based on the response to question 24, and the remaining radiological technologists were as assigned to the non-NPS area group. Basic statistical analysis, cross tabulation analysis, and statistical significance tests were performed.

Knowledge of radiation protection among radiological technologists was evaluated using Welch’s *t*-test considering the sufficiently large sample size, with regionality (NPS areas or non-NPS areas) as an independent variable and the correct answer rates to questions 1– 5 as dependent variables. Similarly, knowledge of radiation disasters was evaluated using Welch’s *t*-test with the correct answer rates to questions 6– 11 as dependent variables. The proportion and number of respondents who chose “Not sure” as an answer to questions 1– 11 were also analyzed. Characteristics of the areas in terms of awareness of radiation protection and radiation disasters were evaluated by using the chi-square test to analyze question 14– 18. Respondents who chose answer 1, 2, or 3 to question 14 were considered to be willing to register with disaster assistance teams and the like, and those who chose answer 1 or 2 to question 17 were considered interested in participating in a radiation disaster-related training course. Respondents who chose answer 1 or 2 to question 18 were considered willing to go to a disaster area in the event of a large-scale disaster (including radiation accidents). IBM SPSS Statistics version 28 (IBM, Armonk, NY) was used for statistical analysis. A *p*-value of <0.05 in a two-tailed test was considered statistically significant in all analyses.

## 4 Results

Responses were received from 148 of the 166 facilities (facility response rate, 89.2%), and from 1391 of the 1835 radiological technologists (response rate, 75.8%). The number of valid responses was 1290 (valid response rate, 70.3%).

### 4.1 Regional differences in knowledge of radiation protection and radiation disasters among radiological technologists

**Fig. 1:**
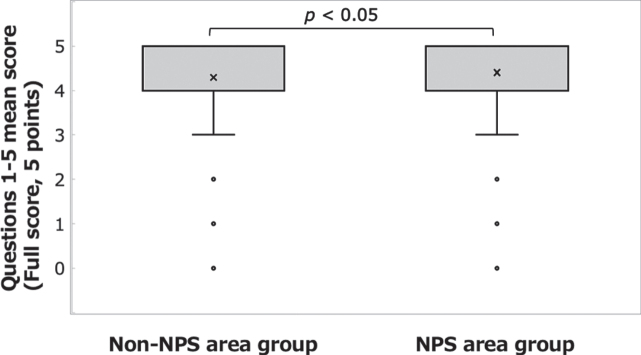
Answers to the questions on basic knowledge of radiation protection. Comparison of the mean scores for questions 1– 5 between the NPS area group and the non-NPS area group.

**Fig. 2:**
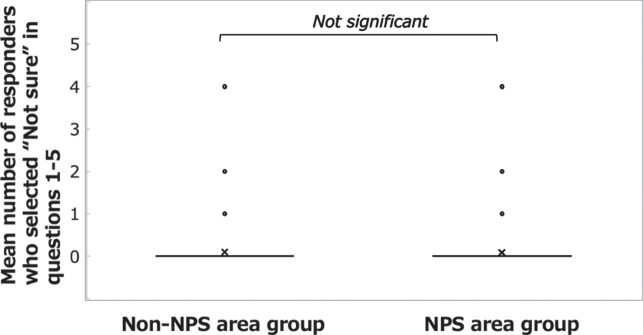
Answers to the questions on basic knowledge of radiation protection. Comparison of the mean numbers of respondents who selected “Not sure” in questions 1– 5 between the NPS area group and the non-NPS area group.

The correct answer rate to questions 1– 5 was high in both the NPS area group (88.0%) and the non-NPS area group (85.8%). The percentages of respondents who answered “Not sure” were 1.8% in the NPS area group and 2.0% in the non-NPS area group. A significant difference was found between the groups in the mean correct answer rate (Fig. 1; Welch’s *t*-test: t(808) = 2.183, *p* = 0.029, *d* = 0.132) but not in the mean number of respondents who selected “Not sure” (Fig. 2; Welch’s *t*-test: t(809) = – 0.576, *n.s.*).

**Fig. 3:**
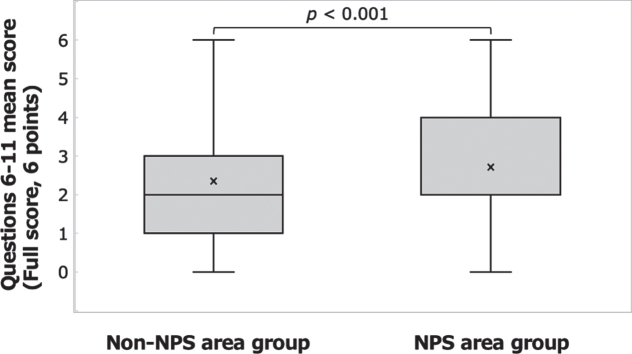
Answers to the questions on basic knowledge of radiation disasters. Comparison of mean scores for questions 6– 11 between the NPS area group and the non-NPS area group.

**Fig.4:**
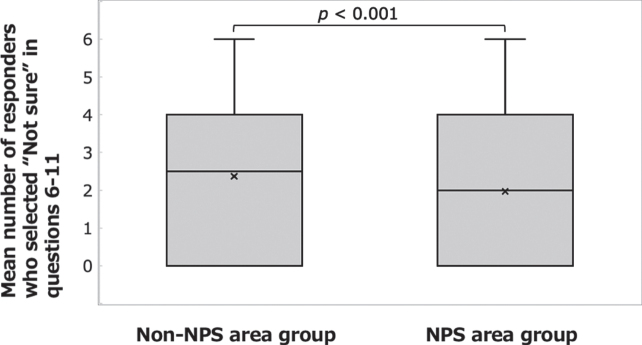
Answers to the questions on basic knowledge of radiation disasters. Comparison of the mean numbers of respondents who selected “Not sure” in questions 6– 11 between the NPS area group and the non-NPS area group.

The correct answer rate to questions 6– 11 was 45.1% in the NPS area group and 39.1% in the non-NPS area group, and the percentage of respondents who selected “Not sure” was 32.8% in the NPS area group and 39.5% in the non-NPS area group. Significant differences were found between the groups in the mean correct answer rate (Fig. 3; Welch’s *t*-test: t(917) = 4.203, *p* <  0.001, *d* = 0.243) and in the mean number of respondents who selected “Not sure” (Fig. 4; Welch’s *t*-test: t(843) = – 3.748, *p* <  0.001, *d* = – 0.224).

### 4.2 Regional differences in awareness of radiation disasters among radiological technologists

1) To question 14, 223 respondents— 145 in the NPS area group (17.0%) and 78 in the non-NPS area group (17.9%)— answered that they were willing to be registered with disaster assistance teams, with no significant difference between the groups (chi-square test: *χ*^2^(1) = 0.168, *p* = 0.682, *φ*= – 0.011).
2) To question 15, 294 respondents— 258 in the NPS area group (30.2% in the group) and 36 in the non-NPS area group (8.3% in the group)— answered that they had previously participated in a course on radiation disasters organized by the national government or a local government. The difference between the groups was significant (chi-square test: *χ*^2^(1) = 79.420, *p* <  0.001., *φ*= – 0.248).
3) To question 16, 161 respondents— 114 in the NPS area group (13.3%) and 47 in the non-NPS area group (10.8%)— answered that they had previously been dispatched for disaster assistance with no significant difference between the groups (chi-square test: *χ*^2^(1) = 1.744, *p* = 0.187, *φ*= 0.037).
4) To question 17, 715 respondents— 477 in the NPS area group (55.9%) and 238 in the non-NPS area group (54.6%)— showed interest in participating in a disaster-related training course, with no significant difference between the groups (chi-square test: *χ*^2^(1) = 0.188, *p* = 0.665, *φ*= 0.012).
5) To question 18, 485 respondents— 321 in the NPS area group (37.6%) and 164 in the non-NPS area group (37.6%)— answered that they would be willing to go to the affected area in the event of a large-scale disaster in the future, if asked by the government or their employers. This difference between the groups was not significant (chi-square test: *χ*^2^(1) = 0.000, *p* = 0.992, *φ*= 0.000). Comparisons of answers to questions 14– 18 between the groups are shown in Table 1.

**Table 1:** Comparison of the awareness of radiation disasters between the NPS area group and the non-NPS area group

Questions	NPS area group		Non-NPS area group		*χ*^2^(1)	*p* value	*φ*
(*n* = 854)		(*n* = 436)	
No. of respondents	%	No. of respondents	%
Q. 14	Respondents who answered that they were willing to register with disaster assistant teams	145	17.0	78	17.9	0.168	0.682	– 0.011
Q. 15	Respondents who answered that they previously participated in a course on radiation disasters organized by the national government or a local government	258	30.2	36	8.3	79.42	<0.001	– 0.248
Q. 16	Respondents who answered that they were previously dispatched for disaster assistance	114	13.3	47	10.8	1.744	0.187	0.037
Q. 17	Respondents who showed interest in participating in a disaster-related training course	477	55.9	238	54.6	0.188	0.665	0.012
Q. 18	Respondents who indicated willingness to go to a disaster area in the event of a large-scale disaster if the government or their employers asked	321	37.6	164	37.6	0.000	0.992	0.000

## 5 Discussion

The National Hospital Organization comprises 166 medical facilities, which provide a variety of medical care, including general medicine and a wide range of specialized care (e.g., cancer treatment and disaster medicine), as well as medical care specific to communities. One characteristic of this organization is that employees move regularly among the facilities, meaning that radiological technologists who belong to this organization can acquire a wide range of work experience. The facility response rate in this study was high (89.2%, 148 of 166 facilities), indicating that there was no regional bias in the responses. The population size was 1835, and the sample size was 1388. This comfortably exceeds the required sample size (385) with a confidence interval of 95% and a margin of error of 5%, indicating that the sample size in this study was statistically valid. The high response rate indicated that radiological technologists of the National Hospital Organization recognized the social importance of this area of study.

The overall percentage of correct answers to the questions on basic knowledge of radiation protection (questions 1– 5) exceeded 80%, indicating that radiological technologists have a high level of knowledge of radiation protection. This is likely to be because radiological technologists developed the necessary skill while preparing for the national examination while in training. Then, after acquiring the national qualification, their knowledge was solidified through training and experience in radiation management during ordinary times. On the other hand, the correct answer rates to the questions on basic knowledge of radiation disasters (questions 6– 11) was below 50%, and some respondents chose “Not sure” as an answer. This might be because there was a difference between awareness of radiation protection in routine practice and that of radiation disasters. This also suggests that any improvement in awareness of radiation disasters after the occurrence of the radiation disaster in Japan is fading, and that fostering knowledge of radiation disasters solely by fulfilling the medical duties is difficult. A growing number of graduates from radiological technologist training schools will have vague memories of past radiation disaster, and a major concern is that awareness of radiation disasters will fade further over time. The percentage of correct answers to questions on basic knowledge of radiation disasters (questions 6– 11) was significantly higher in the NPS area group than in the non-NPS area group. This may be the effect of training and practice in ordinary times, as well as information provided from the national government, local public entities, and other to this group.

On the other hand, because of restrictions in the accounting system for the national budget, it is difficult to establish a system for information dissemination to radiological technologists in non-NPS areas, and this appeared to be a reason for the differences between the groups. This is in a good agreement with the answers to question 15 asking about previous participation in a course on radiation disasters organized by the national government or a local government: the percentage of respondents with previous experience in such course participation was significantly higher in the NPS area group (30.2%) than in the non-NPS area group (8.3%). The difference between the groups was smaller for knowledge than for previous experience in course participation, probably because the acquired knowledge was standardized among radiological technologists as they moved among facilities within the National Hospital Organization.

No significant differences were found between the groups in awareness or experience of disaster assistance activities (questions 14– 18) among radiological technologists at National Hospital Organization facilities. As of the time of the survey, 17.3% of valid respondents were willing registered with disaster assistance teams and the like, irrespective of the area where they worked (question 14). Similarly, 37.6% of valid respondents were willing to go to the affected area in the event of a large-scale disaster (e.g., a radiation disaster), irrespective of the area where they worked (question 18). This difference between willingness to register and willingness to go to a disaster area might be attributable to the level of awareness of disaster assistance teams. To secure human resources for disaster assistance teams, proactive communication with professional associations and academic societies is necessary to increase the visibility of such teams and seek cooperation. Further, 55.4% of all respondents showed interest in participating in a training course on health survey management in event of a radiation accident (question 17), indicating that radiological technologists are willing to participate in such activities and that there is a need for such training courses among them. Satisfying this need can be expected to aid in securing appropriately trained personnel should radiation disasters occur in the future.

This study showed that radiological technologists maintained basic knowledge of radiation acquired during training. They routinely listen to individual patients regarding their concerns about radiation exposure and conduct radiological examinations in routine clinical practice. This would help radiological technologists develop communication ability, share their perspective with patients, listen to them, and ease their anxiety. After the Fukushima nuclear accident, radiological technologists, as they were expected to do, played a role as risk communicators for the local residents [15]. At the time of the Fukushima nuclear accident, 273 radiological technologists were dispatched from all over Japan to the affected area and conducted necessary measurements for 28,704 local residents and contributed to easing their anxiety related to radiation exposure [16]. Also, after the accident, radiological technologists were among the specialists who helped residents to lessen their anxiety through a telephone consultation service established in the area [17]. Such actions by radiological technologists represent a competency model for disaster assistance activities for lessening residents’ anxiety. This study also revealed that, among radiological technologists of the National Hospital Organization, there are potential human resources who had good knowledge and also good awareness of radiation disasters. This means that radiological technologists are appropriate personnel, with their high literacy level, as risk communicators during a radiation disaster.

From the viewpoint of responding to a radiation disaster, the responsible administrative bodies are expected to consider a system that involves radiological technologists who work (or have worked) in the non-NPS area as well as those in the NPS area. Given that the knowledge of radiation disasters was found to depend on the area where they worked, establishment of a nationwide training system, and involvement of the national government and local governments in such a system could lead to effective outcomes including appropriate dissemination of information on the health effects of radiation, elimination of residents’ anxiety, and mitigation of harmful rumors. To prepare for possible radiation disasters in the future, the establishment of an anti-disaster system involving radiological technologists across Japan is necessary.

A limitation of this study is that the subjects were radiological technologists of the National Hospital Organization and did not include those affiliated with private healthcare facilities. Also, data containing non-sampling errors were analyzed in this study.

## 6 Conclusions

A survey of knowledge and awareness of radiation disasters among radiological technologists at National Hospital Organization facilities revealed that their standard of basic knowledge on radiation protection was high, while the levels of basic knowledge on radiation disasters varied depending on whether they worked in NPS areas. This can likely be attributed to the current training system for radiological technologists, and therefore establishment of a nationwide training system can be expected to improve literacy regarding radiation disasters among radiological technologists. They showed high willingness to participate in disaster assistance irrespective of the area where they worked, probably reflecting their risk communication role, which is one their responsibilities. Thus, competencies of radiological technologists represent a competency model for radiation disaster assistance. There is potentially a population of highly motivated radiological technologists, and a system involving them is therefore expected as part of preparation for possible disasters in the future.

## Funding

This work was supported by the Research Project on the Health Effects of Radiation from the Ministry of the Environment, Japan.

## Supplementary Material

Supplementary Material
