# Supplementary material for: Fact-finding survey on the competencies and literacy of radiological technologists regarding radiation disasters
Source: J Xray Sci Technol. 2023 Mar 15;31(2):237–45. doi: 10.3233/XST-221341 (PMC10041441; doi:10.3233/XST-221341)
Supplement: Supplementary Material [file xst-31-xst221341-s001.docx]

**Supplementary material: original questionnaire**

**Request for cooperation in questionnaire survey**

**Fact-finding survey on risk communication in radiation disasters**

1. Which of the following is the correct combination of a unit for radioactivity (A) and a unit for radiation exposure dose that a person receives (B)?
2. I am not sure.
3. A: Bq B: Sv
4. A: J B: Sv
5. A: Sv B: Bq
6. A: Sv B: Gy
7. A: Bq B: J

**Correct answer: 1**

1. Which of the following statements about radiation exposure is correct?
2. I am not sure.
3. The effect of exposure to alpha particles is greatest in the case of external exposure.
4. The effective dose is a measure that estimates deterministic effects.
5. The type and dose of radiation determine its effects on the human body.
6. Effects of exposure to natural background radiation and those of exposure to artificial radiation are different.
7. In internal exposure, radioactive materials accumulate permanently in specific organs.

**Correct answer: 3**

1. Which of the following statements about the evaluation of radiation doses is correct?
2. I am not sure.
3. A background evaluation is necessary.
4. Personal dosimeters are used to detect surface contamination.
5. Ionization-chamber survey meters are used for spectral measurement.
6. GM-counter survey meters are used to evaluate individual doses.
7. NaI(Tl)-scintillation survey meters are used to measure radiation from α-emitting nuclides.

**Correct answer: 1**

1. Which of the following statements about radiation exposure is correct?
2. I am not sure.
3. The average dose of natural background radiation exposure in Japan is higher than that in other parts of the world.
4. Medical exposure accounts for a larger proportion of the annual radiation exposure dose in Japan compared with other parts of the world.
5. Natural background radiation exposure can be reduced to zero by taking precautions in daily life.
6. Air dose rates tend to be lower at higher altitudes due to the strong influence of cosmic rays.
7. Marked increases in cancer incidence and mortality rates have been reported in areas with a high rate of natural background radiation.

**Correct answer: 2**

1. Which of the following statements about radiosensitivity is correct?
2. I am not sure.
3. Radiation exposure can cause permanent infertility in both men and women.
4. Radiation exposure is the only cause of DNA damage.
5. The less radiosensitive cells are, the more actively they divide.
6. Double strand breaks are the only kind of DNA damage caused by radiation.
7. Normal cells always die when exposed to radiation.

**Correct answer: 1**

1. Which of the following correctly describes a Precautionary Action Zone (PAZ) in the nuclear emergency planning zone (of a nuclear power plant)?
2. I am not sure.
3. An area within a radius of approximately 1 km from the nuclear facility
4. An area within a radius of approximately 3 km from the nuclear facility
5. An area within a radius of approximately 5 km from the nuclear facility
6. An area within a radius of approximately 30 km from the nuclear facility
7. An area within a radius of approximately 100 km from the nuclear facility

**Correct answer: 3**

1. Which of the following correctly describes an Urgent Protective action planning Zone (UPZ) in the nuclear emergency planning zone (of a nuclear power plant)?
2. I am not sure.
3. An area within a radius of approximately 1 km from the nuclear facility
4. An area within a radius of approximately 3 km from the nuclear facility
5. An area within a radius of approximately 5 km from the nuclear facility
6. An area within a radius of approximately 30 km from the nuclear facility
7. An area within a radius of approximately 100 km from the nuclear facility

**Correct answer: 4**

1. Which of the following statements about stable iodine intake is correct?
2. I am not sure.
3. It reduces all types of radiation exposure.
4. It should be taken 2 hours or more after exposure.
5. Individuals aged <40 years are the priority group.
6. It reduces internal exposure of the thyroid to radioactive iodine.
7. There is a high likelihood of side effects attributable to anaphylactic shock.

**Correct answer: 4**

1. Which of the following is the correct response to an injured person who has been contaminated with radiation?
2. I am not sure.
3. Bioassay must be performed.
4. Responders must put on personal protective equipment.
5. Decontamination must be prioritized over saving lives.
6. Decontamination must be performed in a specific sequence (wash, wipe off, and then remove one’s clothes).
7. Whole-body counters must be used to evaluate surface contamination.

**Correct answer: 2**

1. Which of the following statements about screening when evacuating from an affected area is correct?
2. I am not sure.
3. Whole-body counters must be used.
4. It is not necessary to inspect vehicles used for evacuation.
5. Only people who need to evacuate when an emergency occurs within the facility premises are subject to screening.
6. Drivers and all passengers must be screened when evacuating by vehicle.
7. Operational intervention level (OIL) 4 is the criteria for decontamination.

**Correct answer: 5**

1. Which of the following statements about the medical system at the time of a nuclear disaster is correct?
2. I am not sure.
3. There is no relationship with local public entities.
4. The Ministry of Health, Labour and Welfare is the responsible administrative body.
5. It is based on the Act on the Regulation of Radioisotopes and other laws.
6. It is applicable only to prefectures where nuclear reactors and related facilities are located.
7. The system consists of primary, secondary, and tertiary medical facilities for conditions related to radiation exposure.

**Correct answer: 4**

1. Please tell us your age as of April 1, 2022.

years old

1. Please indicate your sex.
2. Woman
3. Man
4. Are you currently registered with a disaster medical team (e.g., D-MAT and JMTDR)? Please chose **one**.
5. Yes, I am registered.
6. I am considering registering in future.
7. I am willing to register but cannot get the approval from my family and/or employer.
8. I have no plans to consider registering.

If you chose “1. Yes, I am registered,” please specify the disaster medical team that you are registered with.

1. Have you previously participated in a course on nuclear disasters organized by the national government or a local government?
2. Yes
3. No
4. Have you ever been dispatched as a member of a disaster medical team (including volunteer activities)? Any kind of team is fine. Please choose all that apply.

□ Great Hanshin-Awaji Earthquake Disaster (1995) □ Tokaimura JOC criticality accident (1999)

□ Niigataken Chuetsu-oki Earthquake (2007) □ Iwate-Miyagi Nairiku Earthquake (2008)

□ Great East Japan Earthquake (within Fukushima Prefecture) (2011) □ Great East Japan Earthquake (outside of Fukushima Prefecture) (2011)

□ Kumamoto Earthquake (2016) □ Hokkaido Iburi-Tobu Earthquake (2018)

□ Other (please specify)

1. As part of this study, we are planning to hold an online training course for radiological technologists who are expected to contribute to the management of health surveillance associated with radiation accidents and similar situations. How interested would you be in such a training course?

(Contents of training currently envisioned. Free of charge.)

・Basics of responding to radiation accidents, contamination surveillance, etc.

・Risk communication with residents when a radiation accident occurs.

・Active listening skills in risk communication

1. Very interested in participating.
2. Somewhat interested in participating.
3. No opinion either way.
4. Would rather not participate.
5. Not at all interested in participating.

To those who selected 1 or 2: Please select **all** the topics that you would like to be included in the training course.

□ Medical system at the time of a nuclear disaster　　□ Handling of devices that measure radiation　□ Mental health　□ Risk communication

□ Response to an injured person who is contaminated with radiation　□ Medical care for conditions related to radiation exposure　　　　□　Active listening　　　□ Other (please specify)

To those who selected 4 or 5: Please tell us why you are not interested in participating.

1. If a large-scale disaster (including radiation accidents) were to occur in the future and the government or your employer asked you to go to an affected area, would you want or be willing to go to the disaster area?
2. Yes, very much.
3. Yes, somewhat.
4. Not sure.
5. Probably not.
6. Definitely not.

To those who selected 4 or 5: Please tell us why you would not want or be willing to go to the affected area.

1. Have your or any of the following individuals ever experienced a disaster (e.g., evacuation, temporary relocation, house collapse)? Please select **all** that apply. Disasters include radiation accidents.

□ Me □ My immediate family　　　□ My relatives　　　□ My friends/acquaintances　　　□ None of them

1. Please answer the following questions about your child(ren), if applicable. If you have 4 or more children, please give answers about your three youngest children.

|  | Preschool (Age 0–1) | Preschool (Age 2–3) | Preschool (Age 4–6) | Elementary school | Junior high school | High school | University | Working adult | No applicable child(ren) |
| --- | --- | --- | --- | --- | --- | --- | --- | --- | --- |
| Child 1 | □ | □ | □ | □ | □ | □ | □ | □ | □ |
| Child 2 | □ | □ | □ | □ | □ | □ | □ | □ | □ |
| Child 3 | □ | □ | □ | □ | □ | □ | □ | □ | □ |

1. Please indicate when you obtained your national certification as a radiological technologist.

| Year of national certification as a radiological technologist | | | | | | | |
| --- | --- | --- | --- | --- | --- | --- | --- |
| □ | 1983 | □ | 1993 | □ | 2003 | □ | 2013 |
| □ | 1984 | □ | 1994 | □ | 2004 | □ | 2014 |
| □ | 1985 | □ | 1995 | □ | 2005 | □ | 2015 |
| □ | 1986 | □ | 1996 | □ | 2006 | □ | 2016 |
| □ | 1987 | □ | 1997 | □ | 2007 | □ | 2017 |
| □ | 1988 | □ | 1998 | □ | 2008 | □ | 2018 |
| □ | 1989 | □ | 1999 | □ | 2009 | □ | 2019 |
| □ | 1990 | □ | 2000 | □ | 2010 | □ | 2020 |
| □ | 1991 | □ | 2001 | □ | 2011 | □ | 2021 |
| □ | 1992 | □ | 2002 | □ | 2012 | □ | 2022 |

1. Please indicate which prefecture you are from.

**Hokkaido and Tohoku region**

□ Hokkaido　□ Aomori　□ Iwate　□ Miyagi　□ Akita　□ Yamagata　□ Fukushima

**Kanto and Shinetsu region**

□ Ibaraki　□ Tochigi　□ Gunma　□ Saitama　□ Chiba　□ Tokyo　□ Kanagawa　□ Niigata　□ Yamanashi　□ Nagano

**Tokai and Hokuriku region**

□ Toyama　□ Ishikawa　□ Gifu　□ Shizuoka　□ Aichi　□ Mie

**Kinki region**

□ Fukui　□ ShIga　□ Kyoto　□ Osaka　□ Hyogo　□ Nara　□ Wakayama

**Chugoku and Shikoku region**

□ Tottori　□ Shimane　□ Okayama　□ Hiroshima　□ Yamaguchi　□ Tokushima　□ Kagawa　□ Ehime　□ Kochi

**Kyushu region**

□ Fukuoka　□ Saga　□ Nagasaki　□ Oita　□ Kumamoto　□ Miyazaki　□ Kagoshima　□ Okinawa

1. Please indicate **all** prefectures where you have worked as a radiological technologist from the time of your first employment to March 31, 2011.

□ Not applicable (pre-employment)

**Hokkaido and Tohoku region**

□ Hokkaido　□ Aomori　□ Iwate　□ Miyagi　□ Akita　□ Yamagata　□ Fukushima

**Kanto and Shinetsu region**

□ Ibaraki　□ Tochigi　□ Gunma　□ Saitama　□ Chiba　□ Tokyo　□ Kanagawa　□ Niigata　□ Yamanashi　□ Nagano

**Tokai and Hokuriku region**

□ Toyama　□ Ishikawa　□ Gifu　□ Shizuoka　□ Aichi　□ Mie

**Kinki region**

□ Fukui　□ ShIga　□ Kyoto　□ Osaka　□ Hyogo　□ Nara　□ Wakayama

**Chugoku and Shikoku region**

□ Tottori　□ Shimane　□ Okayama　□ Hiroshima　□ Yamaguchi　□ Tokushima　□ Kagawa　□ Ehime　□ Kochi

**Kyushu region**

□ Fukuoka　□ Saga　□ Nagasaki　□ Oita　□ Kumamoto　□ Miyazaki　□ Kagoshima　□ Okinawa

1. Please indicate **all** prefectures where you have worked as a radiological technologist since April 1, 2011.

**Hokkaido and Tohoku region**

□ Hokkaido　□ Aomori　□ Iwate　□ Miyagi　□ Akita　□ Yamagata　□ Fukushima

**Kanto and Shinetsu region**

□ Ibaraki　□ Tochigi　□ Gunma　□ Saitama　□ Chiba　□ Tokyo　□ Kanagawa　□ Niigata　□ Yamanashi　□ Nagano

**Tokai and Hokuriku region**

□ Toyama　□ Ishikawa　□ Gifu　□ Shizuoka　□ Aichi　□ Mie

**Kinki region**

□ Fukui　□ ShIga　□ Kyoto　□ Osaka　□ Hyogo　□ Nara　□ Wakayama

**Chugoku and Shikoku region**

□ Tottori　□ Shimane　□ Okayama　□ Hiroshima　□ Yamaguchi　□ Tokushima　□ Kagawa　□ Ehime　□ Kochi

**Kyushu region**

□ Fukuoka　□ Saga　□ Nagasaki　□ Oita　□ Kumamoto　□ Miyazaki　□ Kagoshima　□ Okinawa

1. Please select **one** area in which you specialize or are most interested in.
2. General radiography
3. CT examination
4. MRI examination
5. Angiography
6. Nuclear medicine examination
7. Radiation therapy
8. Medical information
9. Radiation protection
10. Disaster medicine
11. Public health
12. Please indicate **all** academic associations that you belong to.
13. The Japan Association of Radiological Technologists
14. Japanese Society of Radiological Technology
15. Japanese Association of Disaster Medicine
16. The Japanese Radiation Research Society
17. Japanese Association for Radiation Accident / Disaster Medicine
18. Japan Health Physics Society
19. Japanese Society of Radiation Safety Management
20. Japanese Society of Public Health
21. Other professional associations related to radiology
22. Please select **all** qualifications that you have obtained from the list below.
23. Radiation Equipment Manager
24. Radiation Protective Manager
25. Radiation Exposure Counselor
26. Certified Disaster Support Radiological Technologist
27. First-class radiation protection supervisor (passed the test, but have not completed the designated training course)
28. First-class radiation protection supervisor (completed the designated training and obtained the license）
29. Other certifications and professional qualifications related to radiology
30. Please tell us whether or not you have experience giving oral presentations at academic conferences (not including study groups or workshops).
31. None
32. Once
33. 2–5 times
34. 6–10 times
35. 11 times or more

This is the end of the questionnaire. Thank you for your cooperation.
